# Sex-specific regulation of follicle-stimulating hormone secretion by synaptotagmin 9

**DOI:** 10.1038/ncomms9645

**Published:** 2015-10-20

**Authors:** Lindsey K. Roper, Joseph S. Briguglio, Chantell S. Evans, Meyer B. Jackson, Edwin R. Chapman

**Affiliations:** 1 Department of Neuroscience, University of Wisconsin-Madison, Madison, Wisconsin, USA; 2 Howard Hughes Medical Institute, Chevy Chase, Maryland, USA; 3 University of Wisconsin-Madison, WIMR 2 Room 5505, 1111 Highland Avenue, Madison, Wisconsin 53705, USA; 4 Molecular and Cellular Pharmacology Program, University of Wisconsin-Madison, Madison, Wisconsin, USA

## Abstract

The anterior pituitary releases six different hormones that control virtually all aspects of vertebrate physiology, yet the molecular mechanisms underlying their Ca^2+^-triggered release remain unknown. A subset of the synaptotagmin (syt) family of proteins serve as Ca^2+^ sensors for exocytosis in neurons and neuroendocrine cells, and are thus likely to regulate pituitary hormone secretion. Here we show that numerous syt isoforms are highly expressed in the pituitary gland in a lobe, and sex-specific manner. We further investigated a Ca^2+^-activated isoform, syt-9, and found that it is expressed in a subpopulation of anterior pituitary cells, the gonadotropes. Follicle-stimulating hormone (FSH) and syt-9 are highly co-localized in female, but not male, mice. Loss of syt-9 results in diminished basal and stimulated FSH secretion only in females, resulting in alterations in the oestrus cycle. This work uncovers a new function for syt-9 and reveals a novel sex difference in reproductive hormone secretion.

## Abstract (web summary)

Pituitary gonadotropes secrete follicle-stimulating hormone (FSH) in both male and female mice. Roper *et al.* show that the synaptotagmin isoform syt-9 is specifically required for FSH secretion, but only in females, revealing a mechanism by which specificity can be encoded in complex secretory cells.

## Graphical abstract

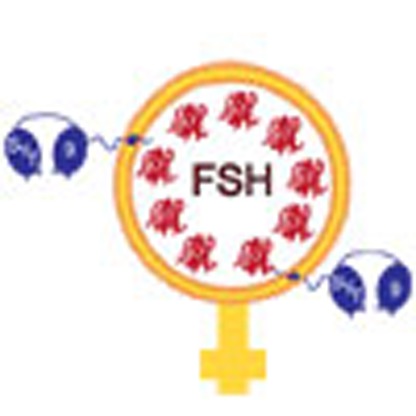

The precise control of peptide hormone secretion from the pituitary is essential for regulating vertebrate physiology and homeostasis, as these hormones control diverse processes including growth, metabolism and reproduction. The anterior pituitary consists of five major cell types, which secrete specific hormones. Gonadotropes are the only pituitary cell type that secrete two hormones: follicle-stimulating hormone (FSH) and lutenizing hormone (LH)^1^; however, they are differentially released^2^, and this difference is necessary for their physiological action. In both males and females, FSH and LH are rhythmically secreted to control reproductive physiology. In males, these hormones drive the synthesis of testosterone and the production and development of sperm. In females, FSH triggers follicle maturation, and a sudden surge in LH drives ovulation. Surprisingly, the differential secretion of FSH and LH are both driven by the pulsatile release of the same hormone, gonadotropin-releasing hormone (GnRH), originating in the hypothalamus. GnRH triggers Ca^2+^ oscillations in gonadotropes^3^,^4^,^5^,^6^ with specific patterns (amplitudes and frequencies) that can differentially trigger the release of FSH and LH. The signalling steps through which GnRH-receptor interactions mobilize intracellular Ca^2+^ are well-understood^7^; however, little is known concerning the Ca^2+^ sensors that ‘decode’ these oscillations to trigger exocytosis.

Hormone secretion is mediated by soluble NSF attachment protein receptor (SNARE) proteins: large dense core vesicles (LDCV) harbour vesicular SNAREs (v-SNAREs) that assemble into four-helix bundles with target membrane SNAREs (t-SNAREs), thus catalysing fusion. Numerous regulatory proteins control SNARE-catalysed fusion, including the synaptotagmin (syt) family of proteins, which have been shown to play crucial roles in the regulation of exocytosis in a variety of cell types including neurons and neuroendocrine cells^8^,^9^,^10^,^11^. Seventeen isoforms of syt have been identified in mammals^12^, and many, but not all, bind to—and are activated by—Ca^2+^ (ref. 13). Moreover, the affinity of syts for Ca^2+^ can differ greatly between distinct isoforms, indicating that syts might be able to differentially integrate Ca^2+^ signals in cells^13^.

In hippocampal neurons, a pHluorin screen revealed that most syt isoforms expressed in brain^14^ recycle, in response to depolarization, with kinetics suggestive of targeting to non-overlapping subsets of LDCVs^15^. Indeed, syt-4 has been shown to regulate the release of brain-derived neurotrophic factor from both axons and dendrites^16^, and syt-10 was proposed to regulate the release of insulin-like growth factor-1 in olfactory neurons^17^. In addition, syt-1/9 and syt-1/7, have been shown to regulate LDCV release in PC12 cells and chromaffin cells, respectively (reviewed in the study by Moghadam and Jackson^10^). The emerging view is that many, if not most, isoforms of syt are LDCV proteins. The pituitary harbours the greatest variety of LDCVs, but little is known concerning the expression and function of syts in this gland.

Syt family members 1, 4, 7 and, as studied here, syt-9, are expressed in most endocrine tissues^10^ and while progress has been made concerning their functions, the role of syt-9 (accession # NM_016908; sometimes referred to as syt-5 (ref. 18) remains controversial and somewhat obscure. In the mouse brain, syt-9 was reported to be a LDCV protein^19^, while another group reported that this isoform was targeted to synaptic vesicles where it acts as a Ca^2+^-sensor for fast release^20^, but this latter finding could not be confirmed in a subsequent study^15^. In addition, in INS-1E cells, knockdown of syt-9 had no effect on basal insulin release, but reduced glucose-stimulated secretion of insulin by 69% (ref. 21); however, changes in insulin secretion were not detected in *in vivo* studies^22^. However, in PC12 cells, it is well-established that syt-9 regulates LDCV secretion^23^,^24^. In addition, analysis of all four syt isoforms expressed in PC12 cells (syt-1, 4, 7 and 9), revealed that each is differentially sorted to LDCVs of different sizes, where they influence the mode of fusion^25^.

Here we show that syt-9 is enriched in both the anterior and posterior lobes of the pituitary. In the anterior pituitary, syt-9 is expressed in a subpopulation of cells, including the gonadotropes. Intriguingly, FSH is highly co-localized with syt-9 in female, but not male, mice, and knockout (KO) of syt-9 in female mice strongly reduces FSH secretion. *Syt-9* KO females also exhibit a prolonged oestrus phase. Finally, we demonstrate that a number of additional syt isoforms are highly expressed, at the protein level, in each lobe of the pituitary. Interestingly, these expression patterns are, in some cases, sex specific. Together these results uncover a novel function for syt-9, provide new insights into sex-specific differences in the control of the endocrine system, and represent a step towards unravelling the molecular mechanisms that underlie the differential secretion of pituitary hormones.

## Results

### Syt-9 expression in the pituitary and other brain regions

**Figure 1:**
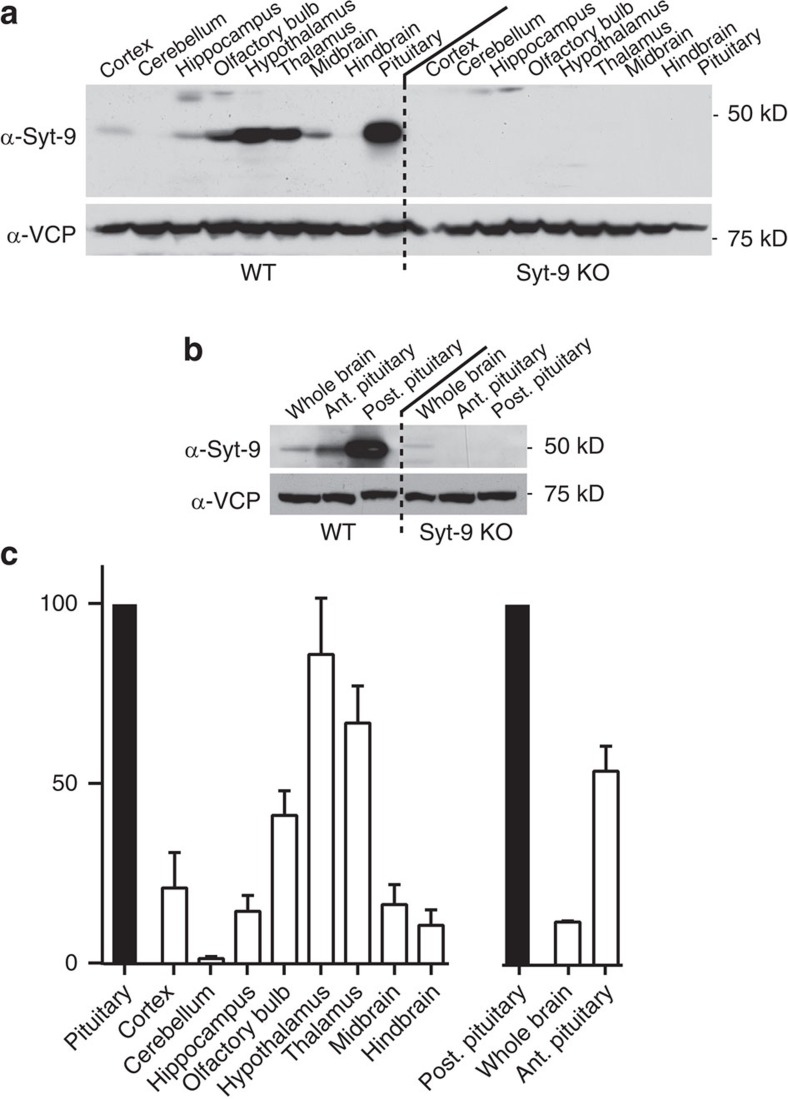
Robust expression of syt-9 in the pituitary. (**a**) Immunoblots of brain tissues probed with an α-syt-9 antibody reveal different expression levels across brain regions, with the highest level of expression in the pituitary gland. (**b**) Syt-9 is expressed in both the anterior and posterior lobes of the pituitary. In **a**,**b** syt-9 was not detected in samples from KO mice, and parallel blots were probed with an α-VCP antibody to confirm equal loading of all samples. (**c**) Quantification of the relative levels of syt-9 expression from **a**,**b**. Expression levels were normalized to samples with the largest signals; whole pituitary for **a** and posterior pituitary for **b**. Representative immunoblots are shown; data were quantified from *N*=3 for **a**, and *N*=2 for **b**. Plotted values are mean±s.e.m.

As outlined in the Introduction, recent studies indicate that many, if not most isoforms of syt, are targeted to LDCVs in neurons; the syts are therefore likely candidates for regulating the release of hormones from the pituitary. To begin to address this issue, an antibody was raised against syt-9 (validation in Supplementary Fig. 1a top two panels), a somewhat controversial isoform that is expressed in both neurons and neuroendocrine cells. This antibody was then used to investigate the expression patterns of syt-9 in different brain regions (Fig. 1; Supplementary Fig. 2); interestingly, the highest expression levels occur in the pituitary (Fig. 1a,c; Supplementary Fig. 2a). The second highest relative expression level of syt-9 was in the hypothalamus, another tissue that is rich in LDCVs. Significant levels of expression were also detected in the olfactory bulb and thalamus, with lower levels in the cortex, hippocampus and midbrain. Syt-9 was not detected at high levels in the cerebellum or hindbrain, or in any brain region obtained from *syt-9* KO mice. Analysis of the individual lobes revealed that syt-9 is expressed at significantly higher levels in the anterior pituitary than in the whole brain, but expression is most robust in the posterior lobe (Fig. 1b,c; Supplementary Fig. 2b).Here we address the function of syt-9 in the anterior pituitary; a separate study will explore the potential role of syt-9 in posterior pituitary hormone secretion.

### Syt-9 is expressed in a subset of anterior pituitary cells

**Figure 2:**
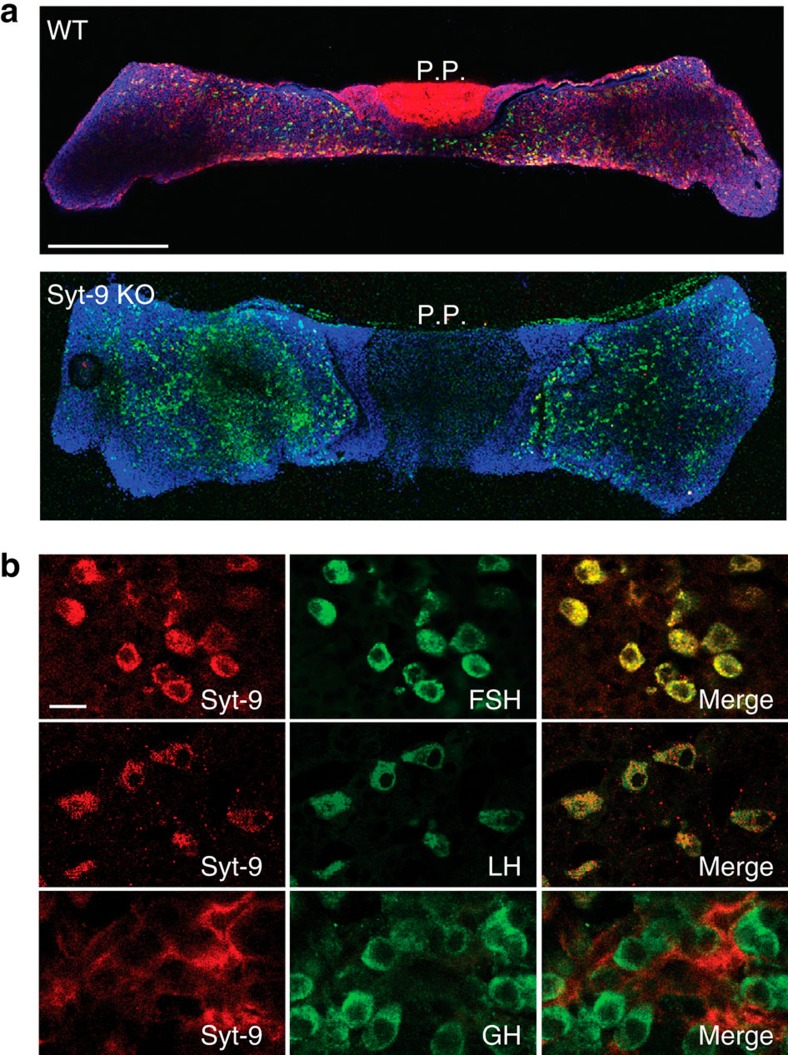
Syt-9 is expressed in gonadotropes but not somatotropes. (**a**) Top panel: coronal section of WT mouse pituitary glands stained with α-syt-9 (red) and α-FSH (green) antibodies; nuclei were stained with DAPI (blue). The posterior pituitary (P.P.) is indicated; the scale bar, 300 μm. Syt-9 is strongly expressed in the posterior pituitary and in cells distributed throughout the anterior pituitary. Bottom panel: same as in the Top panel, but using tissue from *syt-9* KO mice; syt-9 was not detected. (**b**) Higher magnification images of coronal sections from rats revealed syt-9 is expressed in gonadotropes, labelled with FSH and LH, but was not detectable in somatotropes, labelled with GH. Scale bar, 10 μm. Representative images are shown; two independent trials were carried out.

Staining of syt-9 in coronal slices of pituitary glands confirmed robust expression in the posterior lobe and in a subset of cells that are randomly distributed throughout the anterior lobe (Fig. 2a). These sections were co-stained for FSH, to mark gonadotropes within the anterior pituitary, and 4,6-diamidino-2-phenylindole (DAPI) to mark the nuclei of all cells; even at this low level of magnification some degree of co-expression with FSH was apparent. We therefore examined higher magnification images, and in addition to FSH, we stained for LH and growth hormone (GH). These experiments clearly revealed that syt-9 is expressed in gonadotropes, as evidenced by its presence in cells that express FSH and LH. In contrast, syt-9 was not expressed in somatotropes, as shown by the lack of co-localization with GH (Fig. 2b).

### Co-localization of syt-9 with FSH in females

**Figure 3:**
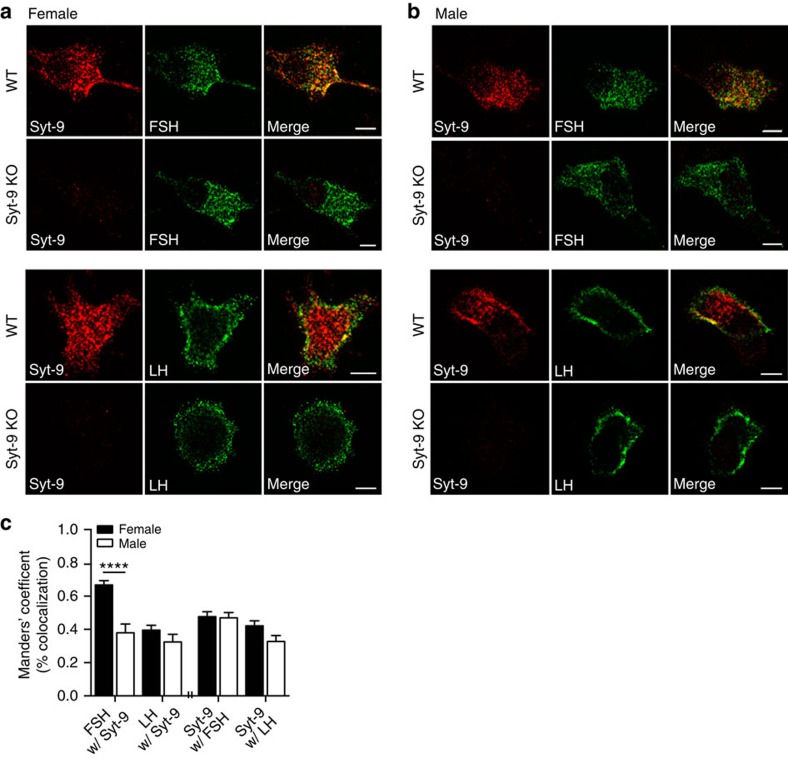
Immunolocalization of FSH and syt-9. (**a**) Pituitary cells isolated from females were co-stained using antibodies against syt-9 (red) and either FSH (upper panel) or LH (green) (lower panel). (**b**) Same as in **a**, but using cells isolated from males. Under each condition, *syt-9* KO cells were included as controls. (**c**) The degree of co-localization of syt-9 with each hormone, as well as the inverse, was quantified. Localization of FSH with syt-9 was significantly higher in female mice, as compared with males. *N*≥20 cells, two independent dissections for each condition. All scale bars, 5 μm. Plotted values are mean±s.e.m. Student’s *t*-test, ****P≤0.001.

To determine the sub-cellular localization of syt-9, and whether it co-localizes with either FSH- or LH-containing vesicles, we turned to primary cultures of dissociated pituitary cells and acquired higher resolution images (Fig. 3a,b). We examined the degree of syt-9 co-localization with each of these hormones, and we also examined the reciprocal: the degree of co-localization of FSH or LH with syt-9 (Fig. 3c). Syt-9 showed some degree of co-localization with FSH in both males and females, ∼48%. Syt-9 also exhibited a low level of co-localization with LH (∼38%) in the gonadotropes of both male and female mice. The reciprocal analysis yielded striking results: in females, 67% of the FSH signal was co-localized with syt-9. In contrast, in males, only 38% co-localization was observed. Finally, ∼36% of LH co-localized with syt-9 in both sexes. These findings reveal a significant sex difference in the sub-cellular localization of syt-9.

### Syt-9 folds t-SNAREs to drive Ca^2+^-regulated membrane fusion

**Figure 4:**
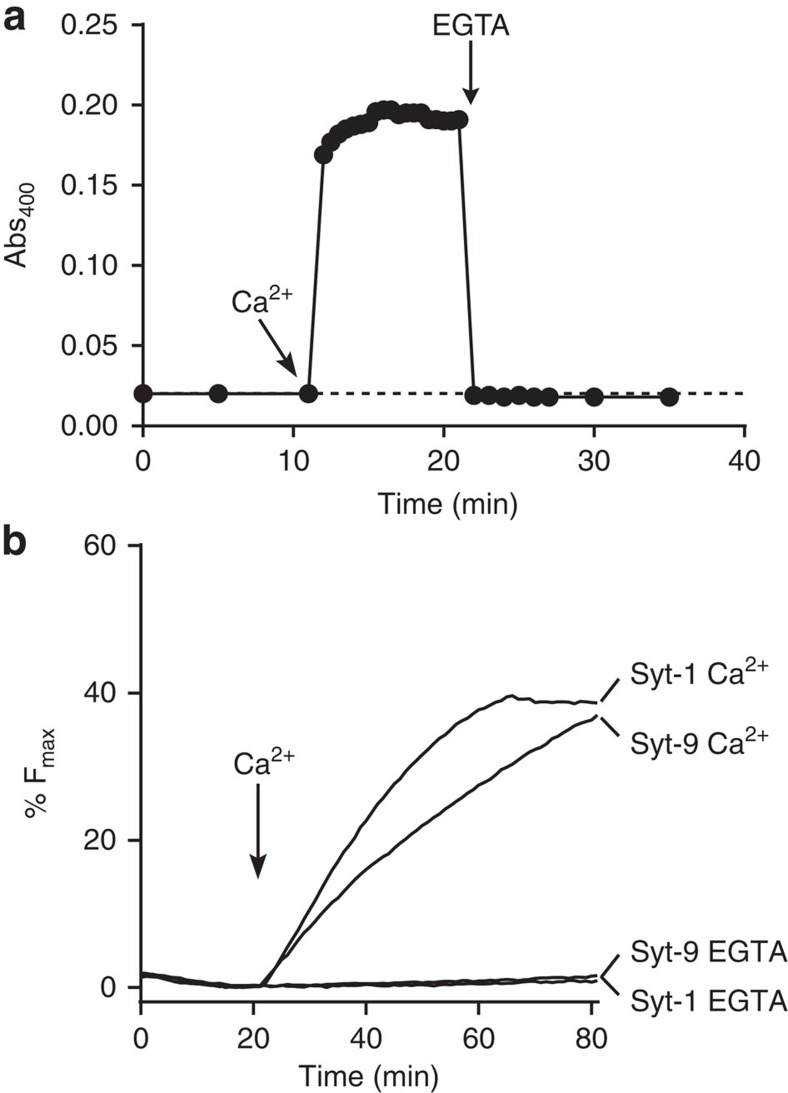
Syt-9 folds t-SNAREs to promote Ca^2+^-regulated membrane fusion. (**a**) A time course of protein-free liposomes turbidity was monitored by measuring the absorbance at 400 nm. On the addition of Ca^2+^, syt-9 mediated liposome aggregation, which was fully reversible by the subsequent addition of EGTA. (**b**) Split t-SNARE *in vitro* membrane fusion assay using reconstituted syntaxin-1A and soluble SNAP-25B. Syt-1 and 9 were able to facilitate membrane fusion in the presence of Ca^2+^, but not in the control EGTA condition. The extent of fusion (%F_max_) was 41.5±4.9 and 41.9±5.3 for syt-1 and syt-9, respectively.

These findings suggest that syt-9 might regulate the exocytosis of gonadotropin-containing LDCVs. In reconstituted systems, Ca^2+^·syt-9 can in fact accelerate SNARE-catalysed fusion^26^; however, in the standard *in vitro* fusion assay, which utilizes pre-assembled t-SNARE heterodimers (syntaxin-1A (syx) and SNAP-25B), fusion is enhanced by simply aggregating v- and t-SNARE vesicles^27^, so it remained unclear as to whether syt-9 can regulate fusion via a more specific mechanism. Indeed, we found that Ca^2+^·syt-9 does in fact aggregate vesicles (Fig. 4a). We therefore utilized a variant of the reconstituted fusion assay in which syx is reconstituted into liposomes and SNAP-25B is added in a soluble form. Under these conditions, syt-1 must first fold SNAP-25B onto syx for fusion with syb-bearing vesicles to occur; aggregation alone has no effect^27^. We found that, syt-9, like syt-1, is able to drive fusion in response to Ca^2+^ in this ‘split t-SNARE’ fusion assay (Fig. 4b). So, syt-9 is a *bona fide* Ca^2+^ sensor that can directly regulate SNARE-catalysed fusion *in vitro*.

### Reduced FSH secretion in female but not male *syt-9* KO mice

**Figure 5:**
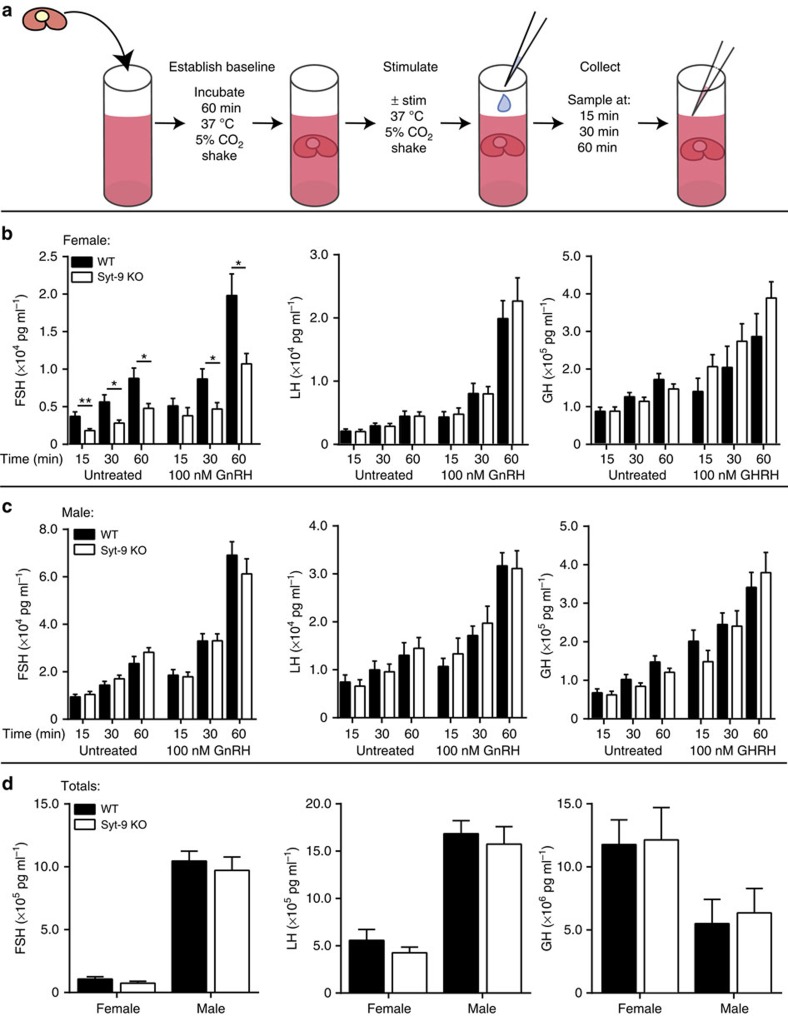
Reduced secretion of FSH in female *syt-9* KO mice. (**a**) Diagram of the *in vitro* stimulation assay used to analyse FSH, LH and GH secretion from intact isolated pituitary glands. Release was stimulated by the addition of 100 nM GnRH (FSH and LH) or 100 nM GHRH (GH). (**b**,**c**) Quantitation of hormone release, as measured using a multiplex ELISA. *Syt-9* KO females exhibited lower levels of FSH release in both unstimulated and stimulated conditions. FSH release in males and LH release in both sexes were unaffected by loss of syt-9. Syt-9 was not detected in cells that release GH, and loss of syt-9 had no effect on GH secretion. (**d**) Total hormone levels in whole pituitary glands: levels of all three hormones are unchanged in *syt-9* KO, as compared with WT, animals. *N*≥10 animals. Plotted values are mean±s.e.m. Student’s *t*-test: *P≤0.05, **P≤0.01.

To determine whether syt-9 regulates FSH or LH release from gonadotropes, hormone secretion from whole dissected pituitary glands (Fig. 5a), from wild type (WT) and *syt-9* KO mice, was monitored with (stimulated) or without (unstimulated) the addition of the relevant tropic hormone. We also monitored GH release as a negative control. Secretion of FSH and LH was stimulated by the addition of GnRH, and the release of GH triggered by adding GH-releasing hormone (GHRH). As shown in Fig. 5b, FSH secretion was significantly reduced in female *syt-9* KO mice, in the stimulated condition, at 30 and 60 min. Interestingly, we also observed reductions in unstimulated FSH secretion at all time points tested; however, LH was unaffected under all conditions. Loss of syt-9 did not affect either FSH or LH secretion in male mice under any conditions tested (Fig. 5c), revealing that the role of syt-9 in FSH secretion is sex specific. Furthermore, GH release was unaffected by loss of syt-9, in both females and males (Fig. 5b,c), which is consistent with the absence of syt-9 in somatotropes. In addition, loss of syt-9 had no effect on the total levels of any pituitary hormones tested (Fig. 5d).

### *Syt-9* KO mice exhibit alterations in the oestrous cycle

**Figure 6:**
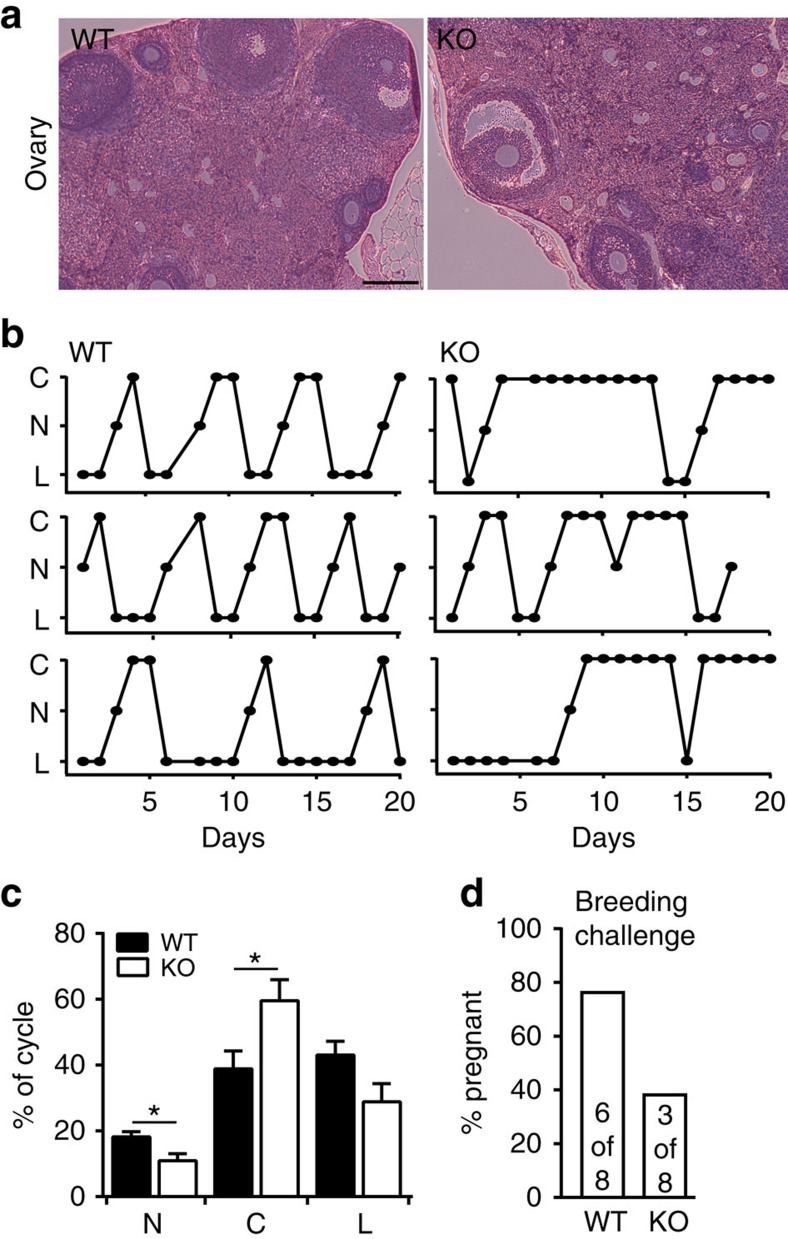
*Syt-9* KO mice exhibit alterations in the oestrous cycle. (**a**) Ovary histology of WT and *syt-9* KO female mice; no major differences were observed. *N*=2. (**b**) Representative oestrous cycles from WT and *syt-9* KO, mice. *N*=3. (**c**) Oestrous cycle data from WT and *syt-9* KO females were averaged and plotted. *Syt-9* KO females exhibited prolonged oestrus phases. *N*=8. (**d**) WT and *syt-9* KO females were paired with WT males for 4 days, separated and monitored for pregnancy and offspring. *N*=8. Plotted values are mean±s.e.m. Student’s *t*-test, *P≤0.05. C, cornified cells; L, leukocytes; N, nucleated cells.

Given the importance of FSH in follicle maturation in the ovary, disruption of FSH secretion could potentially lead to reproductive deficits in *syt-9* KO females. Surprisingly, we found no differences between breeding rates among all combinations of WT and *syt-9* KO mice (Supplementary Fig. 3a). The sex ratios (Supplementary Fig. 3b), as well as the number of offspring in each litter (Supplementary Fig. 3c), were also unchanged. In addition, corpora lutea and follicles of all sizes were present in similar numbers in both the WT and *syt-9* KO animals (Fig. 6a). Serum was collected from female mice in the dioestrus phase of the oestrous cycle as determined by visual inspection of the vaginal opening^28^; however, there were no detectable changes in FSH levels. We then repeated this experiment utilizing oestrous smears^28^,^29^ for more precise timing of the cycle and only found a small but significant decrease in FSH serum levels in *syt-9* KO females (Supplementary Fig. 4b).

While collecting oestrous smears for blood collection, we observed that *syt-9* KO females exhibit subtle, but significant changes in the oestrous cycle. Namely, the oestrus phase of the cycle is longer in the KOs than in WT females (Fig. 6b,c). We therefore re-examined the KO mice for alterations in breeding rates, but this time using a more stringent approach, in which *syt-9* KO females were challenged with a constrained mating period. The mouse oestrous cycle lasts for 4–5 days, so eight WT and eight *syt-9* KO females were individually paired with a male for a period of 4 days; males were then removed. After 18 days, six WT females (75%) were pregnant and gave birth to pups soon after, whereas only three (37.5%) *syt-9* KO females became pregnant and delivered litters (Fig. 6d). Together, these data further support an *in vivo* role for syt-9 in the regulation of FSH release in female mice.

In contrast with *syt-9* KO females, *syt-9* KO males did not exhibit a detectable reproductive phenotype (Supplementary Fig. 3a). Testes histology (Supplementary Fig. 5) and FSH serum levels (Supplementary Fig. 4a) were unaffected in the KOs.

### Differential expression patterns of syt isoforms

**Figure 7:**
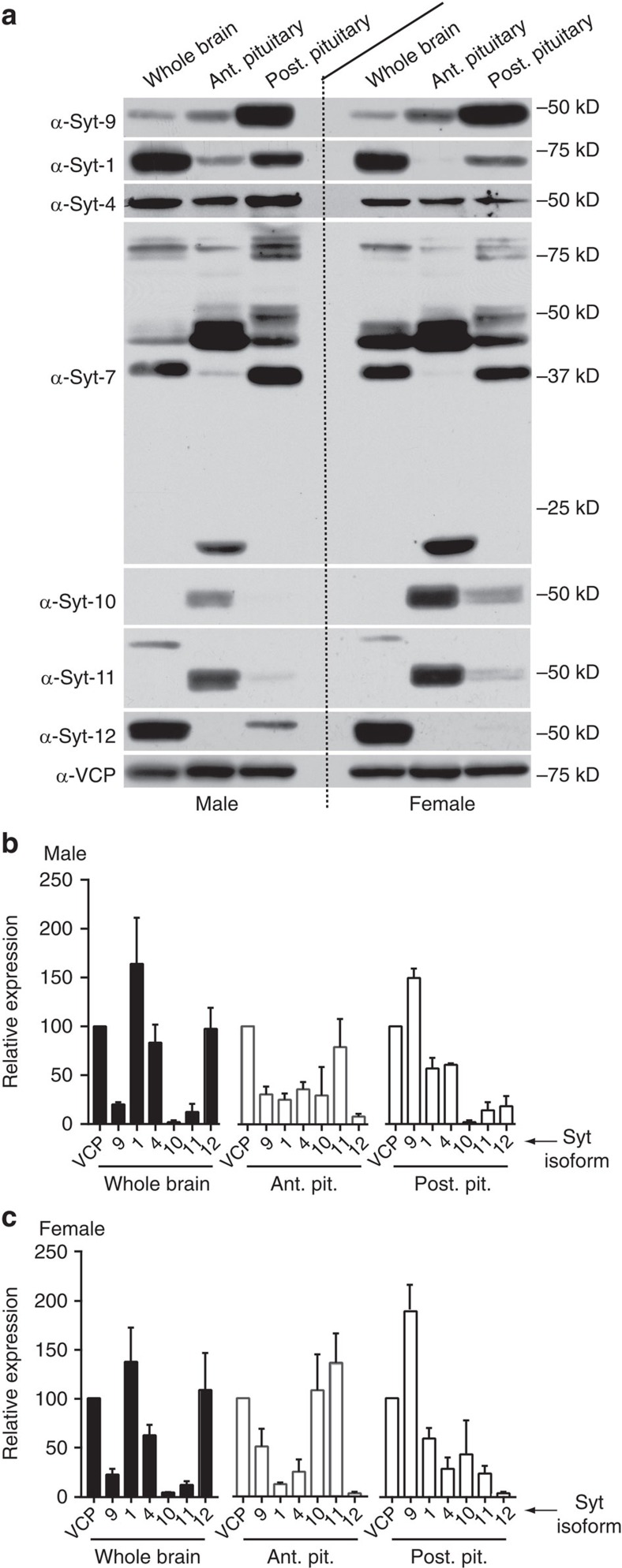
Syt expression levels in the brain and pituitary. (**a**) Immunoblots of whole brain, anterior pituitary and posterior pituitary tissue extracts, from male and female rats. Blots were probed with antibodies against multiple syt isoforms, and with an α-VCP antibody to confirm equal loading. Quantification of expression patterns of multiple syt isoforms, in males (**b**) and females (**c**), normalized to VCP expression. *N*≥2 independent trials. Plotted values are mean±s.e.m.

To further investigate the sex-dependent sorting of syt-9 uncovered above, we compared samples from males and females to determine whether there are sex-specific differences in expression patterns and expressions levels. We also conducted a survey to identify additional syt isoforms that could regulate the secretion other pituitary hormones. Immunoblots of the whole brain, anterior and posterior pituitary extracts were probed with isoform-specific antibodies. For this screen, commercially available antibodies were used to detect syt-1, 4 and 7, and new antibodies were generated against syt-10, 11 and 12 (Supplementary Fig. 1a,b). These experiments revealed that the syt-9 expression pattern was the same in both sexes (Fig. 7; Supplementary Fig. 6). So, while syt-9 displayed sex-specific sub-cellular localization (Fig. 3c), there were no discernible differences in anterior pituitary expression levels in females versus males (Fig. 7). Syt-1 is expressed in the posterior pituitary of both males and females; surprisingly, this isoform was expressed in the anterior pituitary in males only, thus uncovering a novel sex-dependent difference, but in this case at the level of protein expression. Syt-7 is also expressed in the anterior and posterior lobes of the pituitary in both males and females. This isoform is thought to undergo extensive alternative splicing^30^, and striking sex differences in the putative splice variants in each lobe were apparent revealing yet another novel sex-specific difference. Syt-10 and 11 were almost exclusively expressed in the anterior pituitary in both sexes; syt-10 was more abundant in females. Syt-12 also exhibited a marked sex difference: this isoform was detected only in the posterior pituitary of males. Finally, syt-4 is expressed at equal levels in both lobes, and like syt-9 expression is similar in both sexes. We also compared the absolute expression levels of each syt isoform in the two lobes of the pituitary to the levels observed in brain. Syt-7, 10 and 11 are expressed at much lower levels in brain as compared with the anterior pituitary; only syt-1 and 12 were expressed at higher levels in whole brain. Finally, syt-4 was expressed at roughly equal levels in brain versus the pituitary (see also the 2009 study by Zhang *et al.*, where mouse syt-4 was expressed at higher levels in the pituitary than in specific brain regions^31^). Relative expression of all syt isoforms, except syt-7, were quantified using densitometry in males and in females (Fig. 7b,c).

## Discussion

The highly coordinated release of the gonadotropic hormones FSH and LH are crucial aspects of mammalian reproduction. While the differential release of these two hormones has been well-studied, the molecular mechanisms that underlie their secretion remain unknown. The findings that FSH is highly co-localized with syt-9 in female mice (Fig. 3a,c), and that loss of syt-9 leads to reduced FSH release only in females (Fig. 5b), offers the first insight into the Ca^2+^-sensing molecular machinery that regulates differential pituitary hormone secretion, and uncovers a novel sex difference. Because basal FSH release in unstimulated WT females at 60 min equalled the degree of release from female *syt-9* KO glands that had been stimulated with GnRH for 60 min (Fig. 5b), syt-9 appears to play a major role in the Ca^2+^-triggered release of FSH in females, by acting as a Ca^2+^ sensor. Reduced FSH secretion in *syt-9* KO females was also observed under basal conditions, in which release is unlikely to be driven by Ca^2+^. These findings raise the possibility that syt-9 plays additional roles in LDCV trafficking, akin to syt-1, which is thought to contribute to the docking and priming of LDCVs in chromaffin cells^32^. We are currently establishing primary culture systems to directly address this issue using imaging methods that cannot be applied to the whole tissue experiment utilized here.

Because there is some degree of syt-9 co-localization with FSH in males, and with LH in both males and females, it remains possible that subtle alterations occurred but were not measurable in our *in vitro* stimulation assay. It should also be noted that GnRH was added in a single bolus, but *in vivo* this hormone is released from the hypothalamus in a pulsatile manner. Future experiments, in which GnRH is applied in a more temporally controlled manner, might reveal additional alterations in FSH or LH secretion. Finally, it is possible that other factors dominate the regulation of release of LH, to occlude the function of syt-9 on these vesicles. Nonetheless, even the simple addition of GnRH revealed a marked and unexpected sex difference regarding syt-9 control of FSH secretion in males and females.

Numerous mouse models, with deficits in the gonadotropin signalling pathway, have been generated and characterized. Ablation of FSH signalling leads to infertility in females, and has mixed effects on male^33^. Complete loss of FSH, by knocking out the gene for FSHβ^34^, and frame shift mutations in FSHβ in humans^35^, have been shown to arrest follicle development at the primary/preantral stage, leading to infertility. We hypothesize that the partial reduction in FSH secretion observed in *syt-9* KO mice only slows the development of the dominant follicle, thus prolonging the oestrus phase. Indeed, breeding rates were normal, when mice were allowed to breed over a prolonged period. However, if the mating time was restricted, deficits in reproduction were observed (Fig. 6d), and this could be due to diminished serum levels of FSH (Supplementary Fig. 4b).A stronger reproductive phenotype will likely await identification of other additional molecules that drive this residual release. In addition, compensatory changes in the serum half-life of FSH could ameliorate the effect of reduced FSH release, as multiple studies have shown that the ratio of sialic acid to sulfonated N-acetylgalactosamine residues on circulating FSH molecules can drastically alter serum half-life^36^,^37^.

In addition to the sex-specific sub-cellular localization of syt-9, we also uncovered multiple syt isoforms— syt-1, 7, 10 and 12—that exhibited sex-specific expression patterns in the pituitary gland. Further studies are needed to determine the putative sex-specific functions of these differentially expressed isoforms, which might contribute to sex differences in the regulation of other hormones.

Further study of other anterior pituitary syts (that is, 4, 7, 10 and 11) will likely reveal isoforms that regulate the release of additional anterior pituitary hormones, including LH, GH, adrenocorticotropic hormone, thyroid stimulating hormone and prolactin. Moreover, the posterior pituitary, which releases oxytocin and vasopressin, expresses myriad syt isoforms (that is, 1, 4, 7, 9 or 12), but the identity of the isoform(s) that regulate hormone secretion from this tissue remains unknown. Finally, another unresolved question is whether multiple syt isoforms are targeted to overlapping populations of vesicles; in principle, co-targeting of multiple isoforms could contribute to the ability of LDCVs to decode and integrate Ca^2+^ signals.

## Methods

### Animals

Animal use and care was conducted in accordance with the guidelines established by the National Institutes of Health and approved by the Animal Care and Use Committee of the University of Wisconsin-Madison (protocol# M01221). Wistar rats were purchased from Harlan Labs (Indianapolis, IN). *Syt-9* KO mice^20^ were obtained from Jackson Laboratory (Bar Harbor, ME).

### Antibodies and hormones

The rabbit polyclonal α-syt-4 (1:500), α-syt-7 (1:500) and the α-syt-9 (1:500) antibodies used for immunocytochemistry were purchased from Synaptic Systems (Goettingen, Germany). The mouse monoclonal α-valosin-containing protein (VCP) (1:1,000), α-green fluorescent protein (GFP) (1:2,000) and α-his_6_ tag (1:2,000) antibodies were purchased from Abcam (Cambridge, UK). For immunoblot analysis, we used an α-syt-9 (1:250) rabbit polyclonal antibody generated using the C2B domain of the protein as the antigen^25^. Also, α-syt-10 (1:500) and α-syt-11 (1:500) mouse monoclonal antibodies, and an α-syt-12 rabbit polyclonal antibody (1:500), were generated using the C2A domain of each respective syt isoform as the antigen. The α-syt-11 monoclonal antibody is available from Biolegend (San Diego, CA). The mouse polyclonal α-syt-1 antibody hybridoma (mab48; ref. 38 was obtained from Developmental Studies Hybridoma Bank (Iowa City, IA)), and mouse ascites fluid was generated at Covance (Princeton, NJ). Guinea pig and rabbit polyclonal α-FSH, LH and GH antibodies were provided by the National Hormone and Peptide Program (Torrance, CA).

For immunofluorescence, the secondary antibodies were α-guinea pig Alexa 488 and α-rabbit Alexa 647 (1:800) purchased from Life Technologies (Madison, WI). Secondary α-rabbit- or α-mouse-conjugated horseradish peroxidase (H+L) antibodies, used for immunoblot analysis (diluted 1:1,000 or 1:10,000), were obtained from Synaptic Systems.

GnRH (LHRH Salmon) was purchased from Sigma Aldrich (St. Louis, MO), and GHRH (GRF, rat) was purchased from Bachem Chemicals (Bubendorf, Switzerland).

To validate the α-syt-9, -10 and -12 antibodies, HEK cells were transfected with plasmids encoding syt 1–12, fused to GFP, in a pCI vector under a CMV promotor. The N-terminal GFP tags were preceded by a pre-prolactin signal sequence, identical to the pHluorin constructs described in the study by Dean *et al*^15^. Transfections were carried out using the calcium phosphate method. Transfected cells were harvested in a hypotonic lysis buffer (10 mM HEPES, pH 7.4, 2 mM EGTA), and a protease inhibitor cocktail (1 μg ml^−1^ aprotinin, pepstatin and leupeptin, and 1 mM PMSF) using a cell scraper, and homogenized with 20–30 strokes in a dounce homogenizer. Large fragments were removed via centrifugation at 400*g* for 2 min at 4 °C. The supernatant was then subjected to centrifugation at 21,000*g* for 15 min at 4 °C, and the pellet was resuspended in solubilization buffer (50 mM HEPES, pH 7.4, 120 mM NaCl, 1% Triton X-100, 0.5% cholic acid plus the protease inhibitor cocktail). Samples were again subjected to centrifugation at 21,000*g* for 15 min at 4 °C, and the resulting supernatant was diluted in SDS sample buffer (125 mM SDS, 62.5 mM Tris, 10% sucrose, 10% BME and bromophenol blue) boiled for 5 min, and subjected to SDS–PAGE and immunoblot analysis.

The α-syt-11 mouse monoclonal antibody was validated against recombinant his_6_-tagged cytoplasmic domains of syts 1–12.

### Recombinant proteins

Complementary DNA was provided as follows: syx and synaptobrevin-2 (syb) were from J.E. Rothman (New Haven, CT), SNAP-25B was from M.C. Wilson (Albuquerque, NM), syt-1, -3 and -4 were from T. Sudhof (Palo Alto, CA; we repaired the G374 mutation in syt-1 via substitution with a glycine), syt-2 and syts 5-11 were from M. Fukuda (Sendai, Japan) and syt-12 was from C. Thompson (Baltimore, MD). The cytoplasmic domain of syt-1 (96–421) was subcloned into a pGEX vector to generate a GST fusion protein. The cytoplasmic domains of syts 1–12 (syt-2, 139–423; syt-3, 290–569; syt-4, 152–425; syt-5, 218–491; syt-6, 143–426; syt-7, 134–403; syt-8, 97–395; syt-9, 104–386; syt-10, 223–501; syt-11, 150–430; and syt-12, 114–421), and full-length versions of all three SNAREs (syx, syb and SNAP-25B) were subcloned into a pTrcHis vector to generate his_6_ fusion proteins.

Bacterial cultures were grown overnight at 37 °C to an OD_600_ of 0.6. Recombinant protein expression was induced with 400 μM IPTG for 5 h at 37 °C. Bacteria were collected by centrifugation (3,700*g* for 15 min at 4 °C) and resuspended in 50 mM HEPES pH 7.4, 100 mM NaCl (GST purification buffer) or 25 mM HEPES pH 7.4, 100 mM KCl, 20 mM imidazole, 10% glycerol and 5 mM 2-mercaptoethanol (his_6_ purification buffer). Bacteria were sonicated in the presence of the protease inhibitor cocktail and solubilized with 1% Triton X-100 for 2 h at 4 °C. Insoluble material was removed by centrifugation (39,700*g* for 25 min at 4 °C) and the supernatant was incubated overnight, at 4 °C, with glutathione- or Ni-Sepharose beads, for GST or his_6_ fusion proteins, respectively. Beads were washed twice with 50 vol of their respective purification buffer containing an additional 1 M NaCl and 10 μg ml^−1^ DNAse/RNAse, followed by two more washes in purification buffer without added salt or nucleases. For GST fusion proteins, proteins were cleaved from beads using thrombin (2 h at room temperature (RT)); thrombin was inactivated with PMSF. His_6_ fusion proteins were eluted from beads using 500 mM imidazole; imidazole was removed by extensive dialysis. Purification of his_6_-tagged full-length syx and syb fusion proteins was carried out using the same protocol as above except that 1% Triton X-100 was included in all buffers through the first two washes; 1% octyl glucoside was included in the final washes. Proteins were subjected to SDS–PAGE and Coomassie blue staining; protein concentrations were determined using a BSA standard curve.

### Protein extraction and immunoblot analysis

For immunoblotting, harvested tissue samples were homogenized in phosphate-buffered saline (PBS) plus protease inhibitors, on ice, with a glass–teflon homogenizer, ∼12 strokes, at 900 r.p.m. Samples were solubilized with 1% Triton X-100, rotated at 4 °C for 1 h and then centrifuged at 18,000*g* at 4 °C for 20 min. The supernatant was collected and the protein concentration was determined using a bicinchoninic acid protein assay. Samples were diluted in SDS sample buffer and 20–40 μg of protein was subjected to SDS–PAGE and immunoblot analysis. For all blots, immunoreactive bands were visualized using the SuperSignal West Pico Chemiluminescent substrate from Thermo Scientific (Waltham, MA). Immunoblot data were quantified by densitometry using Image J; data were normalized as detailed in the figures.

### Isolated pituitary cell culture

Pituitary glands were dissected in ice-cold dissection buffer (HBSS without Ca^2+^ or Mg^2+^, 10 mM HEPES, 10 mM glucose and 500 μg ml^−1^ BSA) and digested with 20 U ml^−1^ of papain at 37 °C, in 5% CO_2_, for 20 min. The digested tissue was then washed three times with plating medium (DMEM with 10% foetal bovine serum plus 100 IU ml^−1^ penicillin and 100 μg ml^−1^ streptomycin), triturated and plated on glass coverslips, coated with 0.25 mg ml^−1^ poly-D-lysine, for 48 h in an incubator at 37 °C with 5% CO_2_ before fixation.

### Tissue slice preparation

To stain coronal slices of the pituitary, 10–12-week-old animals were perfused with cold PBS followed by cold 4% paraformaldehyde in PBS. The pituitary was then removed, post fixed in 4% paraformaldehyde for 30 min at RT, suspended in 5% agarose and cut into 100-μm slices using a vibratome.

### Immunocytochemistry

Pituitary coronal slices were washed three times in TBS, permeabilized for 30 min in PBS plus 0.5% Triton X-100 and blocked overnight at 4 °C in PBS plus 0.1% Triton X-100 and 4% BSA. Primary antibodies were added and incubated overnight at 4 °C. The stained slices were washed three times in PBS plus 0.1% Triton X-100, and incubated overnight at 4 °C with secondary antibodies. Slices were washed in the same buffer three times and immediately imaged.

Cultured pituitary cells were fixed in 4% paraformaldehdye in stabilization buffer (5 mM PIPES, 1 mM NaH_2_PO_4_,125 mM NaCl, 5 mM KCl, 2 mM MgCl_2_, 1 mM EGTA, 5 mM glucose, 400 nM sucrose) at 37 °C for 15 min, washed three times with TBS and permeabilized in PBS with 0.5% Triton X-100 for 15 min at RT. Cells were blocked with 4% BSA in PBS for 2 h at RT and incubated with primary antibodies overnight at 4 °C. Cells were then washed three times with PBS plus 0.1% Triton X-100 and incubated with secondary antibody for 1 h at RT. Finally, cells were washed four times with PBS plus 0.1% Triton X-100 and mounted in ProLong Gold (Life Technologies, Carlsbad, CA).

### Image acquisition and co-localization analysis

Images of pituitary coronal slices were collected at RT using a Leica TCS-LSI Macro Confocal with a monochrome CCD camera and × 2 macro objective. Magnified slice images were acquired at RT on an Olympus FV1000 laser scanning confocal microscope, with PMT-based detection and a × 40/0.80 water objective lens. Images were acquired with Olympus Fluoview software using identical laser and gain settings for all samples. Isolated pituitary cell images were collected on the same confocal microscope, but using a × 100/1.49 numerical aperture (NA) oil objective lens. For each WT and *syt-9* KO staining pair, cells were fixed and stained in parallel, imaged with Olympus Fluoview software, again using identical laser and gain settings, and then identically processed in ImageJ (initial background subtraction, Gaussian filtered (*σ*=1.0)). Co-localization was measured according to the Manders’ overlap coefficient. This coefficient was calculated using the ImageJ plugin, JACoP^39^. As a result of the staining heterogeneity in the pituitary cells, signal thresholds were manually adjusted for each image to include all recognizable puncta. All vaginal smear images were collected on an Olympus CKX41 Inverted Microscope with a DP21 stand-alone camera and × 20/0.4 NA objective.

### *In vitro* pituitary stimulation

Ten to 12-week-old animals were killed using CO_2_; intact pituitary glands were then removed and placed in DMEM with a protease inhibitor cocktail. All female mice were killed during the dioestrus phase of the cycle, as determined by visual inspection^28^. All incubations were carried out at 37 °C, 5% CO_2_, with constant shaking. After 60 min, the medium was replaced with fresh medium with or without 100 nM GnRH or 100 nM GHRH. Fifty microlitres samples were taken at 15, 30 and 60 min, and were immediately frozen or assayed for secreted hormones. Pituitary glands were also collected following stimulation to measure total hormone levels. Each gland was placed in cold homogenization buffer (50 mM Tris pH 7.5, 150 mM NaCl, 0.002% Tween 20) with protease inhibitor cocktail, and homogenized with 20 strokes in a glass–teflon homogenizer followed by rotation at 4 °C for 15 min. Samples were then centrifuged at 12,000*g* for 15 min and the supernatant was collected. All hormone measurements were performed with a Milliplex MAP rat pituitary magnetic bead panel (RPTMAG-86 K; Millipore, Billerica, MA) on a Luminex Magpix (Austin, TX) with Milliplex Analyst software.

### Statistical analysis

Statistical significance was determined via the two-tailed unpaired Student's *t*-test. All data shown represent mean±s.e.m. Significance is denoted as follows: *P≤0.05, **P≤0.01, ***P≤0.005, and ****P≤0.001.

### Ovary and testes histology

Freshly dissected ovaries and testes from mice ≥12 weeks of age were fixed in formalin and embedded in paraffin. About 10-μm slices were then cut using a microtome, and stained with haematoxylin and eosin. Samples were imaged using an Olympus CKX41 inverted microscope with a DP21 camera and × 20/0.4 NA objective.

### Serum collection and hormone measurements

Blood was collected via tail tip bleeding from mice ≥12 weeks of age. Blood from female mice was collected during the dioestrus phase of the cycle between 1000 and 1100 hours. Blood was allowed to clot for 30 min at RT followed by centrifugation for 10 min at 1,000*g*. Serum was collected and frozen immediately; later, serum was thawed and assayed using a Milliplex MAP rat pituitary magnetic bead panel, on a Luminex Magpix with Milliplex Analyst software.

### Oestrous cycle analysis

The stage of the oestrous cycle was assessed daily by visual inspection^28^ or from vaginal smears^28^,^29^. Oestrous smears were mounted on slides and imaged immediately using an Olympus CKX41 inverted microscope with a × 20/0.4 NA objective and a DP21 camera.

### Breeding

Breeding challenges were performed by pairing eight individually housed WT and *syt-9* KO females each with a WT male for 4 days. Mating pairs were then separated, and females were monitored to determine if they were pregnant and gave rise to litters.

### Liposome aggregation assay

All lipids were purchased from Avanti Polar Lipids. 15% PS (1,2-dioleoyl-sn-glycero-3-phospho-l-serine; phosphatidylserine (PS)), 30% PE (1-palmitoyl-2-oleoyl-sn-glycero-3-phosphoethanolamine; phosphatidylethanolamine (PE)) and 55% PC (1-palmitoyl-2-oleoyl-sn-glycero-3-phosphocholine; phosphatidylcholine (PC)), in chloroform, were dried under a stream of nitrogen. Films were rehydrated in buffer (50 mM HEPES pH 7.4, 100 mM NaCl). Protein-free liposomes were generated by extruding the mixture through a 50-nm filter.

The cytoplasmic domains of syt-9 (1 μM) and 0.1 mM lipid were mixed with buffer (50 mM HEPES pH 7.4, 100 mM NaCl) and the absorbance was monitored at 400 nm using a DU 730 Life Science UV/Vis Spectrophotometer (Beckman Coulter). After 10 min, 1 mM Ca^2+^ was added and the turbidity was observed for an additional 10 min; 2 mM EGTA was subsequently added to the mixture.

### *In vitro* fusion assay

Syb vesicle lipid composition was 15% PS, 27% PE, 55% PC, 1.5% NBD-PE (1,2-dipalmitoyl-sn-glycero-3-phospho-ethanolamine-N-(7-nitro-2-1,3-benzoxadiazol-4-yl) and 1.5% Rho-PE (N-(lissamine rhodamine B sulfonyl)-1,2-dipalmitoyl-sn-glycero-3-phosphoethanolamine). Syx vesicle lipid composition was 25% PS, 30% PE and 45% PC. SNARE-bearing vesicles were prepared by rapid dilution and dialysis. The indicated lipid mixture, in chloroform, was dried under a stream of nitrogen. Films were rehydrated in 25 mM HEPES pH 7.8, 100 mM KCl, 10% glycerol, 1 mM dithiothreitol (reconstitution buffer) plus 1% octyl glucoside and protein (syx or syb), and subsequently diluted with reconstitution buffer for vesicle formation. Vesicles were dialyzed against reconstitution buffer and were isolated using an Accudenz step gradient. Vesicles were collected and subjected to SDS–PAGE and Commassie blue staining to confirm protein incorporation^40^.

A scaled-down version of the ‘split t-SNARE’ fusion assay was used in this study^41^. Fusion was monitored via dequenching of NBD fluorescence, due to loss of Forster resonance energy transfer (FRET), using a Synergy HT multi-detection microplate reader (Bio-Tek). Briefly, 0.5 μl syb vesicles, 4.5 μl syx vesicles and 7 μM soluble SNAP-25B were added to wells containing 1 μM of the cytoplasmic domain of syt-1 or syt-9, 0.2 mM EGTA and reconstitution buffer. After a 20-min incubation at 37 °C, 1 mM Ca^2+^ (final concentration) was added to each well and the reaction was monitored for 60 additional minutes. After each experiment, the maximum fluorescence signal was determined by the addition of 20 μl of 2.5% n-dodecyl-β-d-maltoside; this value was used to calculate the %F_max_.

## Additional information

**How to cite this article:** Roper, L. K. *et al.* Sex-specific regulation of follicle-stimulating hormone secretion by synaptotagmin 9. *Nat. Commun.* 6:8645 doi: 10.1038/ncomms9645 (2015).

## Supplementary Material

Supplementary Information Supplementary Figures 1-6
