# Supplementary material for: Sex-specific regulation of follicle-stimulating hormone secretion by synaptotagmin 9
Source: Nat Commun. 2015 Oct 20;6:8645. doi: 10.1038/ncomms9645 (PMC4620939; doi:10.1038/ncomms9645)
Supplement: Supplementary Information — Supplementary Figures 1-6 [file ncomms9645-s1.pdf]

Supplementary Figure 1

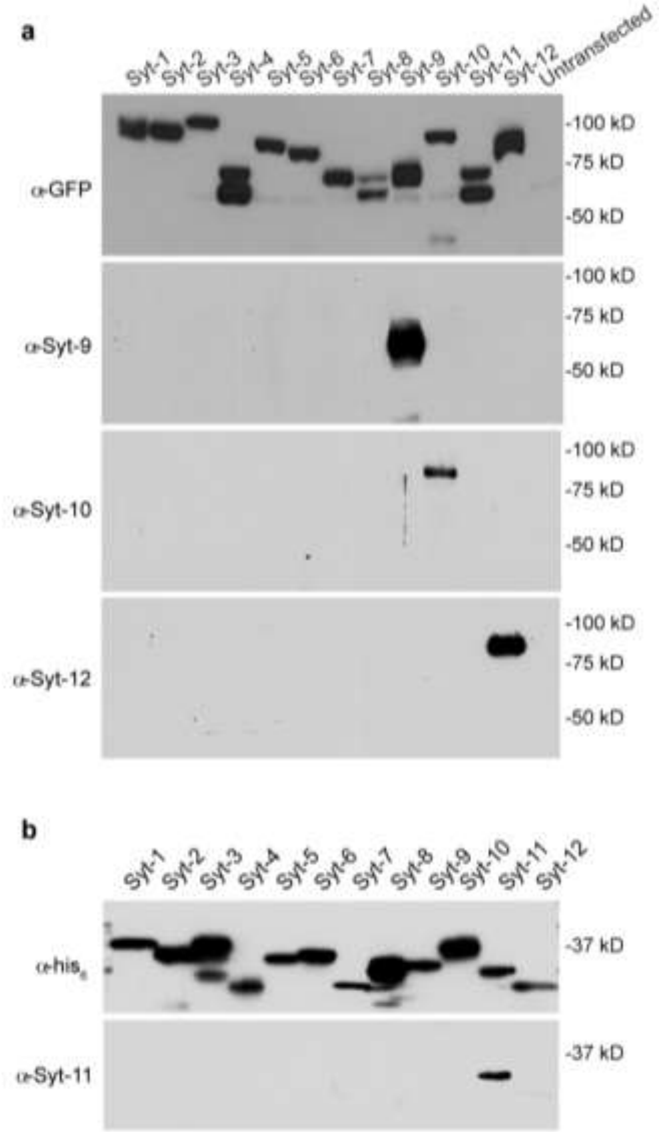

Supplementary Fig. 1. **Validation of novel α-syt isoform specific antibodies.** (a) Immunoblot analysis of HEK cells that had been transfected with GFP-tagged syts 1-12; un-transfected cells were included as a control. *Upper panel*: Similar levels of transfected fusion protein were present in each sample, as revealed using an α-GFP antibody. *Lower panels*: Identical blots were also probed with α-syt-9, 10, and 12 antibodies; in each case the antibody was isoform-specific. (b) Validation of an α-syt-11 antibody using recombinant proteins. *Upper panel*: Fifty ng of each his_6-tagged cytoplasmic domain of syt (isoforms1-12) was subjected to SDS-PAGE and immunoblot analysis using an α-his_6 tag antibody. *Lower panel*: Identical blots were probed with the α-syt-11 antibody; the antibody recognized only syt-11.

Supplementary Figure 2

a

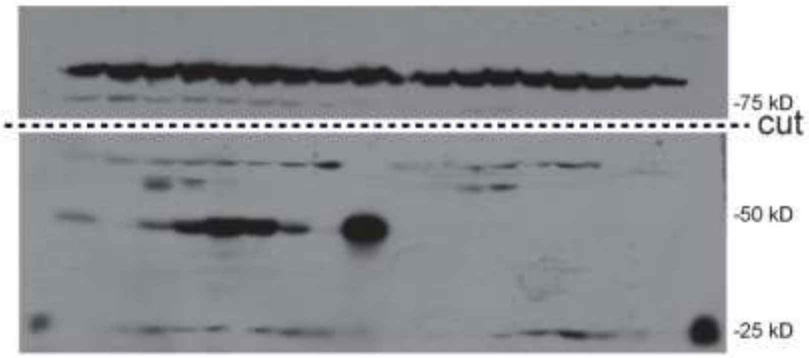

b

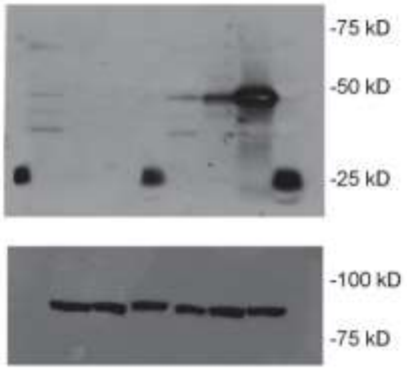

Supplementary Fig. 2. **Full blots utilized in Figure 1a and b.** The original blot from Fig. 1a was cut into two pieces just below the 75 kD marker. The top portion of the blot was probed with the α-VCP antibody and the bottom portion was probed with an α-syt-9 antibody. In the syt-9 blot, a small degree of cross reactivity was observed. In Fig. 1c, data from N = 3 blots were analyzed. (b) Two original blots from Fig. 1b; in this case syt-9 and VCP were probed in separate blots; the syt-9 blot was intact, but the VCP blot was cut to reduce the amount of antibody used. In Fig. 1c data from N =2 blots were analyzed.

Supplementary Figure 3

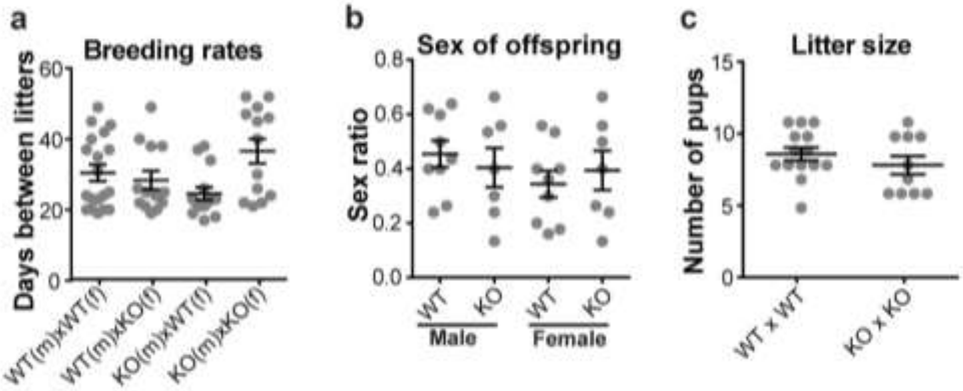

Supplementary Fig. 3. **Analysis of *syt-9 KO* mouse husbandry.** (a) Breeding rate, (b) sex of offspring, or (c) litter size, were unaffected in *syt-9 KO* animals. N = 7-19 litters. Plotted values are means ± SEM. Student's t-test.

Supplementary Figure 4

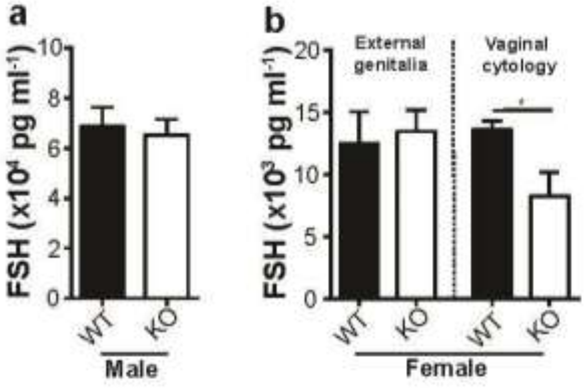

Supplementary Fig. 4. **Serum levels of FSH in *syt-9 KO* mice.** (a) FSH serum levels in male mice were unchanged. N ≥ 18. (b) FSH serum levels in diestrous females, assessed by visual inspection of external genitalia, were unchanged. N ≥ 24. However, when a more stringent assessment of estrous cycle timing, as determined via daily estrus smears, was utilized, a small but significant decrease in FSH serum levels in *syt-9 KOs* was revealed. N = 4. Plotted values are means ± SEM. Student's t-test *P ≤ 0.05.

Supplementary Figure 5

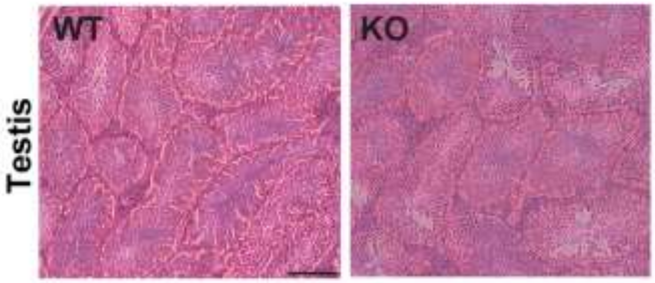

Supplementary Figure 5. **Histology of *syt-9 KO* testes.** (a) Sections of testis from WT and *syt-9 KO* mice were stained and embedded in paraffin as detailed in Supplementary Methods. No significant morphological differences were observed. N = 2; scale bar = 200 µm.

Supplementary Figure 6

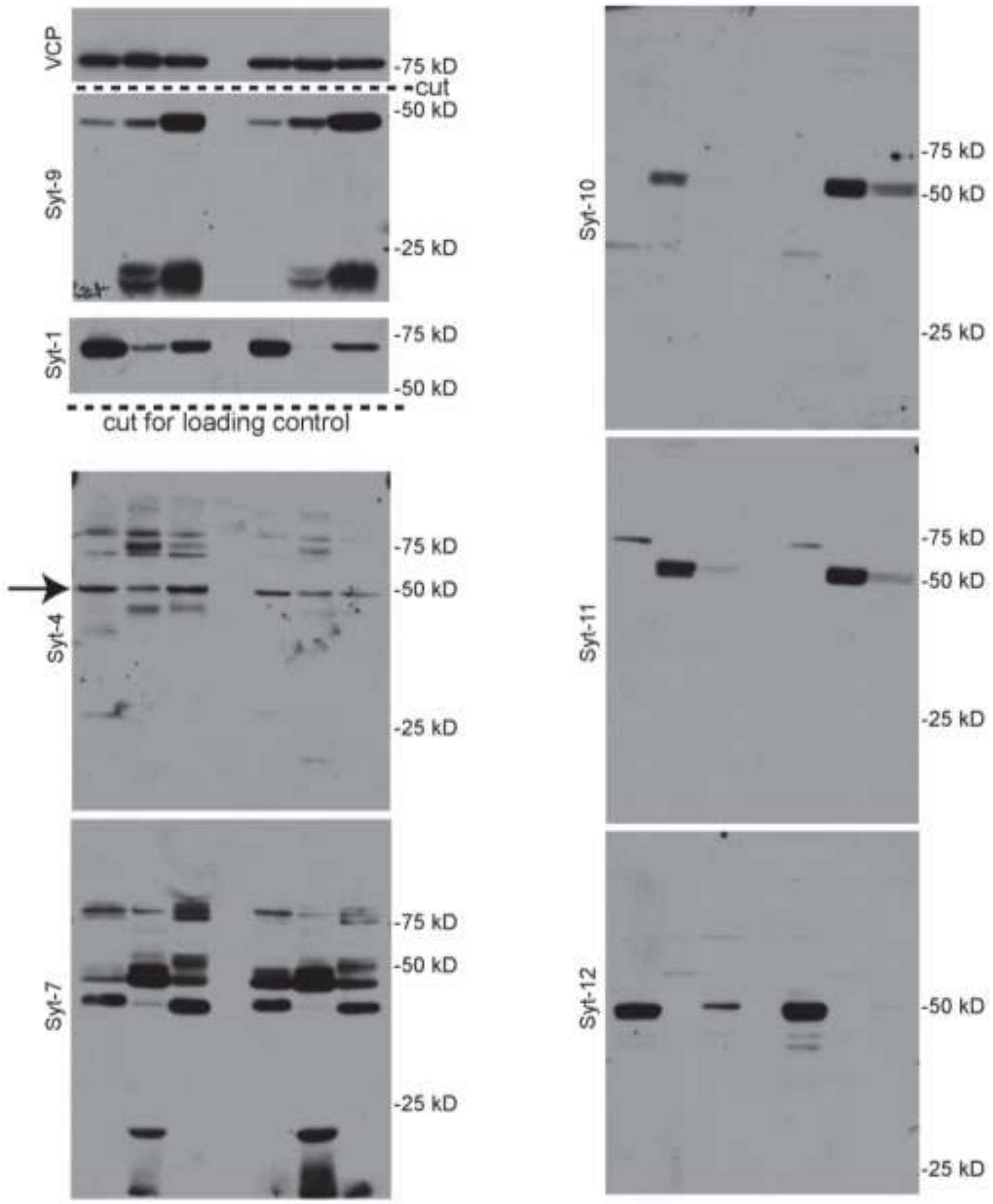

Supplementary Figure 6. **Full blots utilized in Figure 7.** The syt-1 and -9 blots were cut into 2 pieces (as illustrated by the dashed line) for loading controls so the entire blot could not be shown. The α-syt-4 antibody exhibits cross reactivity, so the syt-4 band is indicated with an arrow.
